# Apical root resorption during orthodontic treatment with aligners? A retrospective radiometric study

**DOI:** 10.1186/1746-160X-9-21

**Published:** 2013-08-14

**Authors:** Elena Krieger, Thomas Drechsler, Irene Schmidtmann, Collin Jacobs, Simeon Haag, Heinrich Wehrbein

**Affiliations:** 1 Department of Orthodontics, Medical Centre of the Johannes-Gutenberg-University Mainz, Augustusplatz 2, 55131 Mainz, Germany; 2 Private practice, Wilhelmstraße 40, 65183 Wiesbaden, Germany; 3 Institute of Medical Biostatistics, Epidemiology and Informatics, University Medical Centre of the Johannes-Gutenberg-University Mainz, Obere Zahlbacher Str. 69, 55131 Mainz, Germany

**Keywords:** Apical root resorption, Thermoplastic appliances, Aligners, Orthodontic, Tooth movement, Anterior crowding

## Abstract

**Introduction:**

Objective of this study was to investigate the incidence and severity of apical root resorptions (ARR) during orthodontic treatment with aligners.

**Materials and methods:**

The sample comprised 100 patients (17–75 years of age) with a class I occlusion and anterior crowding before treatment, treated exclusively with aligners (Invisalign®, Align Technologies, Santa Clara, CA, USA). The following teeth were assessed: upper and lower anterior teeth and first molars. Root and crown lengths of a total of 1600 teeth were measured twice in pre- and post-treatment panoramic radiographs. Afterwards, relative changes of the root length during treatment were calculated by a root-crown-ratio taking pre- and post-treatment root and crown lengths into consideration. A reduction of this ratio was considered as a shortening of the initial root length. Additionally, tooth movements of the front teeth were assessed by lateral cephalograms and the 3-dimensonal set up of each patient.

**Results:**

All patients had a reduction of the pre-treatment root length with a minimum of two teeth. On average 7.36 teeth per patient were affected. 54% of 1600 measured teeth showed no measurable root reduction. A reduction of >0%-10% of the pre-treatment root length was found in 27.75%, a distinct reduction of >10%-20% in 11.94%. 6.31% of all teeth were affected with a considerable reduction of >20%. We found no statistically significant correlation between relative root length changes and the individual tooth, gender, age or sagittal and vertical orthodontic tooth movement; except for extrusion of upper front teeth, which was considered as not clinical relevant due to the small amount of mean 4% ARR.

**Conclusions:**

The present study is the first analyzing ARR in patients with a fully implemented orthodontic treatment with aligners (i.e. resolving anterior crowding). The variety was high and no clinical relevant influence factor could be detected. A minimum of two teeth with a root length reduction was found in every patient. On average, 7.36 teeth per patient were affected.

## Introduction

Root resorptions (RR) appear to be multifactorial and might be a combination of mechanical effects, a genetic disposition and an individual biological variability [1]. They are described as a permanent loss of tooth structure from the root apex and the clinical manifestation among orthodontic patients is highly variable [2]. Former publications referred that orthodontically treated patients were more likely to gain severe apical root resorption (ARR) [3-5].

Regarding influence factors, Weltman et al. [1] reported in their systematic review comprehensive orthodontic treatment could cause increased incidence and severity of RR, and heavy forces might be particularly harmful. Therefore, they recommended the use of light forces especially for intrusion of anterior teeth [1]. The incidence of ARR in incisors seems to be higher than in other teeth and more severely [6-10]. Recent studies investigated the incidence and severity of ARR in patients treated with multibracket appliances, assessing the ARR in bone beam computed tomography (CBCT) [11,12]. They reported about most significant ARR and highest frequencies in incisors and first molars [11] or a significant association between the upper dentition and anterior teeth with the degree of root shortening [12]. Factors like archwire sequencing, bracket prescription, and self-ligation seemed not affecting ARR [1]. Also seemed previous trauma or tooth morphology unlikely being a causative factor [1].

Considering genetic factors influencing the incidence of ARR, Sehr et al. [13] reported that local rather than systemic or genetic factors seemed to have predisposed the subjects of their sample to ARR. Other publications of animal experimental or human studies found various candidate genes potentially directly linked to the development of severe root resorption [2,8,14]. Harris et al. [15] reported about a proportion of the hereditary component of 60–80% for ARR in patients undergoing orthodontic treatment.

Regarding the incidence of ARR and orthodontic treatment with removable thermoplastic appliances (aligners) there are only three publications available [16-18]. At first, a relation between aligner-therapy and RR was described in the case report of Brezniak and Wasserstein [16]. A 25-year-old patient sustained apical root resorption (ARR) of the upper incisors during treatment with aligners. Also 2008, Barbagallo et al. [17] compared in a prospective study the effects of removable thermoplastic appliances with light and heavy orthodontic forces on premolar cementum with a treatment period of eight weeks. They reported that the aligner group had similar but slightly greater RR than the light-force group or approximately six times greater than the untreated control group [17]. Then, Sombuntham et al. examined 2009 aligners and RR in an animal experimental study [18]. Clear plastic appliances were inserted and bonded to the residual dentition in rats for a maximum of seven days [18]. They found histological changes of the periodontal ligament and initial superficial RR [18].

Therefore, this is the first study examining the incidence and severity of ARR in patients, who underwent a fully implemented orthodontic treatment with aligners. A further aim of the present study was to evaluate, if any external factor like age or gender could be found that had an influence on ARR.

## Material and methods

### Study design and subjects

The sample comprised 100 healthy patients, who were treated exclusively with removable thermoplastic appliances, i.e. aligners (Invisalign®, Align Technologies, Santa Clara, CA, USA). The treatment was performed by one specialist for orthodontics in one private practice. The sample was selected retrospectively from a larger pool of patients by using patient documentation and based on the following inclusion criteria: class I occlusion with an anterior crowding (arch length discrepancy <8 mm), which had to be completely corrected. Also, radiographs had to be available (i.e. pre- and post-treatment panoramic radiographs and lateral cephalograms). Exclusion criteria were evidence of root resorptions on the pre-treatment panoramic radiographs, presence of severely dilacerated roots or endodontically treated teeth. Patients requiring other orthodontic systems, extraction therapy or any surgical treatments were also excluded.

In this practice, the radiographs were taken as a standard procedure for diagnostics before and at the end of active orthodontic treatment. No additional radiograph was conducted for this investigation.

To increase the aligner retention, additional attachments (i.e. tooth-coloured composite material) were applied as recommended by the manufacturer.

The anterior crowding was resolved by IPR (interproximal enamel reduction) and/or protrusion of the anterior teeth, based on the individual patient and determined by the orthodontist, depending on the initial overjet (protrusion) or shape of the tooth (IPR). The mean conducted IPR was 0.33 mm (Min. 0 mm, Max. 0.5 mm) per proximal contact.

Due to the primarily treatment issue, i.e. resolvement of anterior crowding, all upper and lower incisors and canines were assessed. Because of their use as anchorage during treatment, the first upper and lower molars were also assessed. Thus, a total of 1600 teeth were evaluated (400 upper, 400 lower incisors; 200 upper, 200 lower canines; 200 upper, 200 lower first molars).

### Radiographic examinations

All radiographs were taken with the same device. The measurement of the dental panoramic radiographs took place by using an electronic digital caliper rule (Studenroth GmbH Präzisionstechnik, Germany), calibrated in concordance with VDI/VDE/DGQ (Association of German Engineers/Association for Electrical, Electronic & Information Technologies/German Society for Quality) guideline m2618 Sheet9.1 to an accuracy of 0.01 mm. All root and crown measurements were taken twice in each radiograph and measured by one examiner blinded in a stochastic sequence.

**Figure 1:**
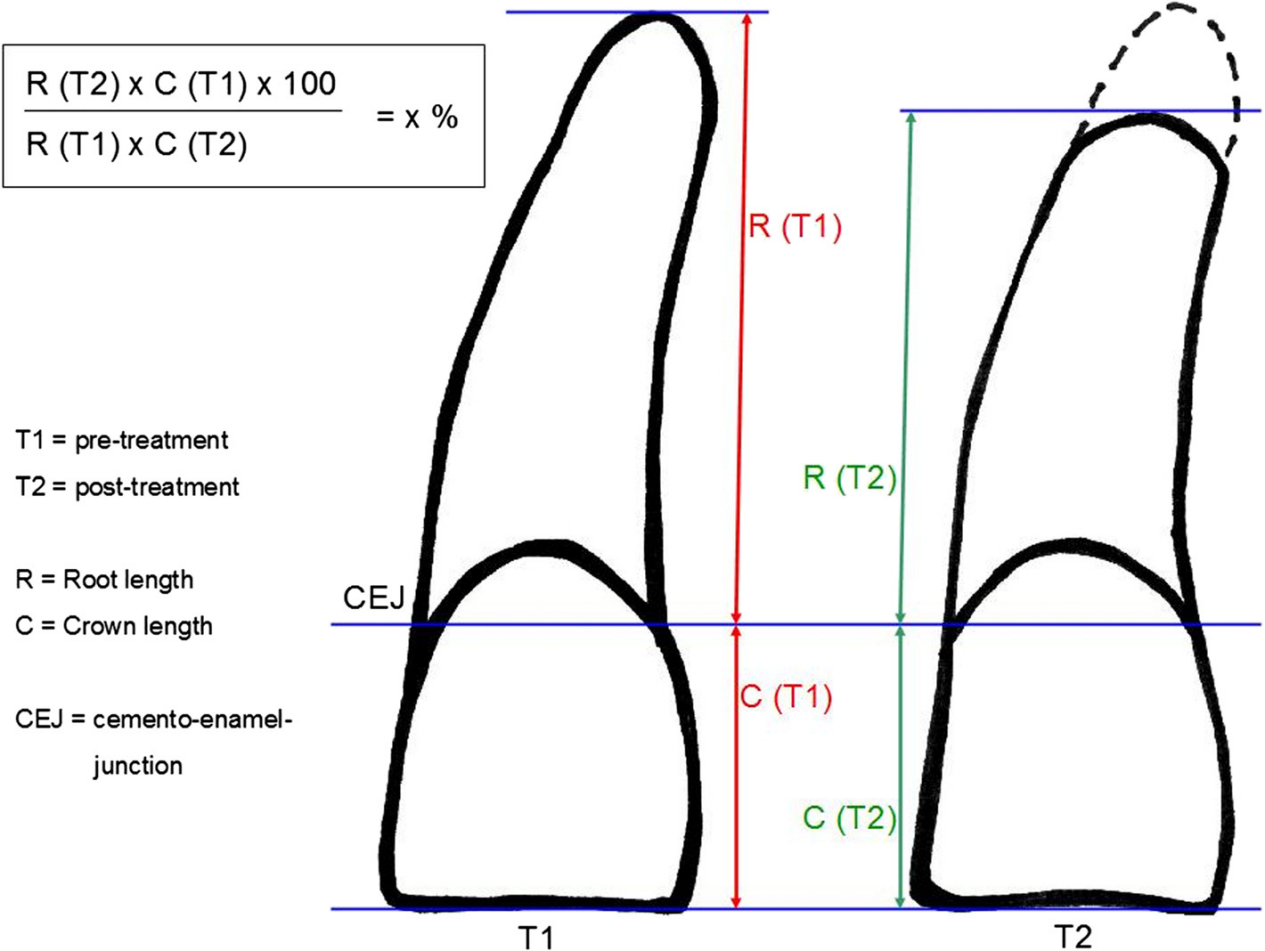
**Measurement of the panoramic radiographs: root and crown lengths and the calculation-formula for the relative change of the root-crown-ratio (RCR) during orthodontic treatment: post-treatment RCR relative to pre-treatment RCR (rRCR) in percentage; (CEJ = cemento-enamel junction) **[7,10]**.**

To assess the pre- and post-treatment root length the following variables were measured under fourfold magnification on the basis of Fritz et al. [7] and Linge and Linge [10]: a symmetric cross was defined by two connecting lines. At first the long axis of each tooth was constructed by a line from the width of the incisal edge to the apex. The second line was defined by the connection of the mesial and distal cemento-enamel-junction. The crown length was declared as the distance between incisal edge and cemento-enamel-junction (on the long axis). The root length was defined as the distance between cemento-enamel-junction and apex (Figure 1).

### Analysis of the radiographs and virtual images

The panoramic radiographs were as usual not standardized. Thus, errors like different projections or magnifications cannot be eliminated. But the crown-root length relation remains stable in different radiographs [7]. Therefore, we used the relation between the vertical root and crown length for further examination, which was described in another investigation [7] (Figure 1). Taking under consideration the pre–and post-treatment root and crown lengths, we calculated the individual root-crown-ratio (RCR) of each tooth and therefore the relative changes of RCR (rRCR) by a formula shown in Figure 1. An rRCR of 100% indicates no change of the pre-treatment root length relative to the post-treatment root length. A decrease of rRCR indicates a reduction of the root length during treatment.

To assess the real amount of orthodontic tooth movement in the front, we superimposed pre- and post-treatment lateral cephalograms. Sagittal and vertical alterations of the tooth position were measured in millimeters, as described in a previous investigation [7]. Vertical movement of the apex was categorized in extrusion or intrusion and sagittal movement in protrusion or retrusion. Tooth movement ≥1 mm was defined as relevant to the possible changes of RCR.

To assign the planned tooth movement of the front teeth, we examined sagittal and vertical movements in the individual 3-dimensional virtual images of each patient (ClinCheck®, Align Technologies, Santa Clara, CA, USA). Previous studies showed a high accuracy between the virtual images and the treatment outcome [19,20]. Therefore, the images of before and at the end of treatment were compared and measured by a grid, set with the lowest adjustable scale ranges of 1 mm.

### Statistical analysis

Data analysis and data collection were performed using the SPSS software program (Statistical Package for Social Science) for Windows Version 18.0 (Inc., Chicago, II, USA) and the SAS software program, version 9.2 (Cary, NC).

The averages of the two measurements were used to compute RCR and the changes in RCR. We computed absolute and relative frequencies for categorical variables. Quantitative measurements are described by mean and standard deviation, minimum, maximum, median and quartiles. For testing changes in RCR we used log (RCR_t2i_/RCR_t1i_) = log (RCR_t2i_)–log(RCR_t1i_) and performed a Hotelling T^2^ test. In order to assess a possible dependence of change in RCR on tooth movement we fitted a mixed linear model to log (RCR_t2i_/RCR_t1i_).

A Hotelling T^2^ test was performed to show significant changes in RCR during treatment. It gave a p-value of 0.0837. Therefore, we fitted a linear mixed model to log(rRCR) including the following parameters as fixed effects: tooth position, intrusion, extrusion, retrusion, protrusion of upper jaw, intrusion, extrusion, retrusion, protrusion of lower jaw. We compared this model to a model only including an intercept term and a random patient effect.

### Method error

Before the main measurements the examiner was calibrated by measuring ten different radiographs five times in random order and a minimum of two weeks in between the measurements.

For each tooth position and for both crown and root length we determined the components of variance –for inter-individual variation σ^2^_A_ and the error variance σ^2^_e_., this yields the intra-class-correlation ICC = σ^2^_A_/(σ^2^_A_ + σ^2^_e_).

## Results

### Calibration experiment

The intra-class-correlation ranged from 0.845 (tooth 41) to 0.992 (tooth 33) for the crown measurements and from 0.929 (tooth 23) to 0.998 (tooth 43) for the root measurements, which indicated a high reproducibility and reliability.

### Patient age, gender, treatment time and tooth position

The patients were on average 37.7 (17–75) years of age, 37 males and 63 females with a mean treatment time of 19.8 months. On average, 32 aligners as well as 6 attachments were applied in the upper and lower jaw.

Analyzing these factors and the influence of the position of the tooth it turned out that none of these external factors was statistically significant correlated to the incidence of ARR (Table 1).

**Table 1:** Statistical analysis of the influence factors (n. s. = not significant)

**Influence factors**	**Statistical significance**
Age	n. s.
Gender	n. s.
Individual tooth	n. s.
Change in RCR during treatment	n. s.
Sagittal tooth movement incisors	n. s.
Vertical tooth movement incisors	n. s.
Except: Extrusion of upper front teeth	p = 0.0184

### Planned and real amount of tooth movement

The planned tooth movement was analyzed by examination of the images (ClinCheck®) before and the end of treatment. In most of the cases a retrusion of the incisors (52% of all 100 patients) was planned in the upper jaw, mostly without vertical movements (43%). Instead, in the lower jaw a protrusion (49% of the cases) and intrusion (65%) was planned.

Regarding the real amount the distribution is shown in Table 2. The mean tooth movement of the incisors ranged in the upper dentition from 0.52 mm (intrusion) up to 1.02 mm (retrusion), with minimum and maximum values of 0 mm up to 4 mm. In the lower jaw the mean values ranged from 0.52 mm (intrusion) to 0.82 mm (protrusion), also with minimum and maximum values of 0 mm to 4 mm. When looking at the F-tests for the individual parameters, it turned out that only the effect extrusion of the upper incisors can be considered as significant (p = 0.0184) (Table 1). On average, patients with extrusion of the upper incisors had only 96.1% of the rRCR of those patients without; i. e. extrusion of the upper incisors leads to more decrease in post-treatment RCRs than treatments without extrusion.

**Table 2:** Distribution of vertical and sagittal orthodontic tooth movement of upper and lower incisors of every patient (n = 100), measured after superimposition of the pre- and post-treatment lateral cephalograms; mean, minimum and maximum values [mm], SD = standard deviation

	***Protrusion***		***Retrusion***		***Extrusion***		***Intrusion***	
	**Upper**	**Lower**	**Upper**	**Lower**	**Upper**	**Lower**	**Upper**	**Lower**
**Mean**	0.81	0.82	1.02	0.56	0.80	0.68	0.52	0.52
**SD**	1.08	1.21	1.22	1.06	1.08	1.05	0.78	0.78
**Min.**	0.00	0.00	0.00	0.00	0.00	0.00	0.00	0.00
**Max.**	4.00	5.00	4.00	4.00	4.00	3.00	3.00	3.00

### Incidence and severity of ARR

**Figure 2:**
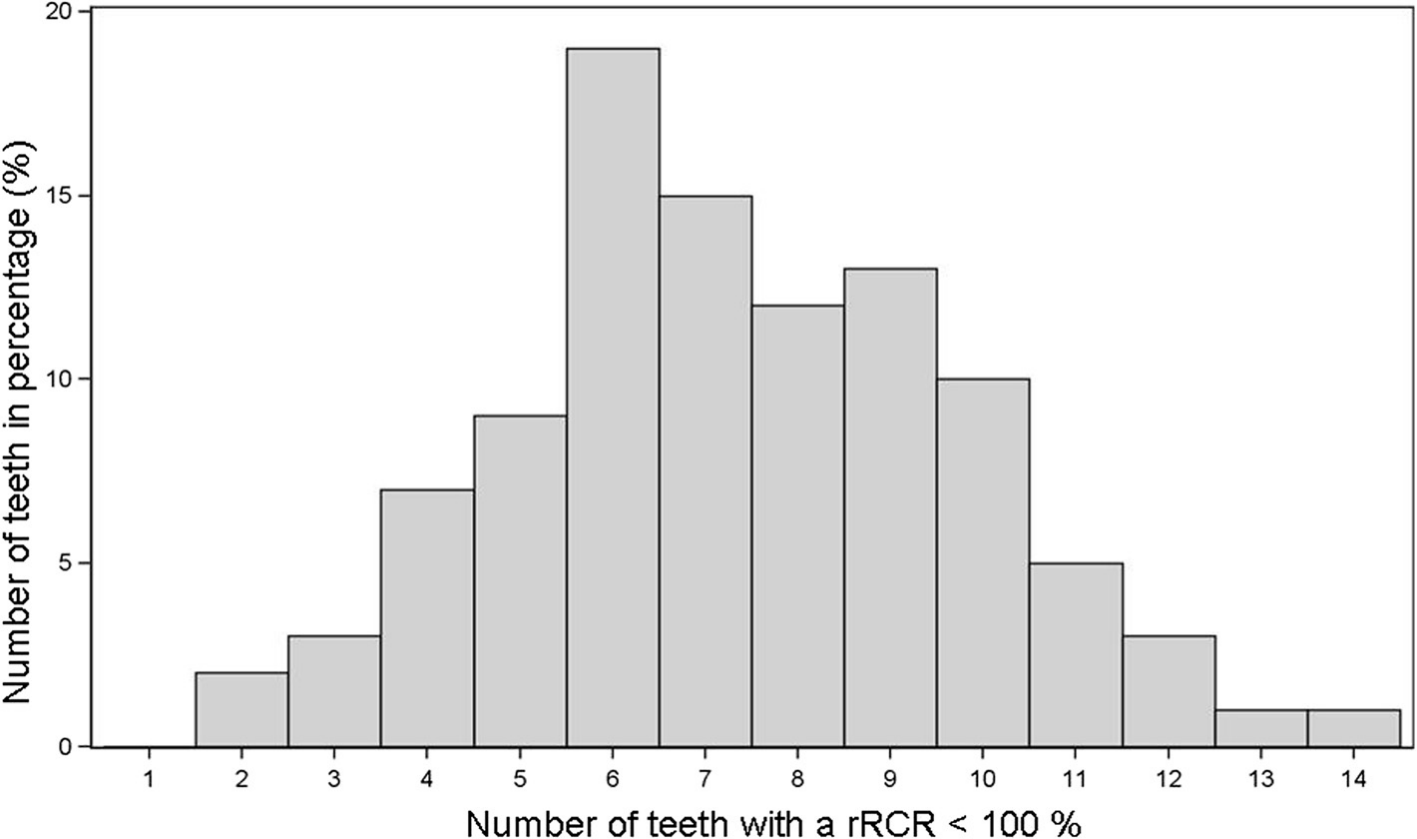
Distribution of the amount of the affected teeth per patient (n = 100).

All patients had a minimum of two teeth affected with a reduction of the root length (rRCR < 100%), on average 7.36 teeth per patient (Figure 2). 61% of all patients had a minimum of one tooth with a 20% root length reduction after treatment.

**Figure 3:**
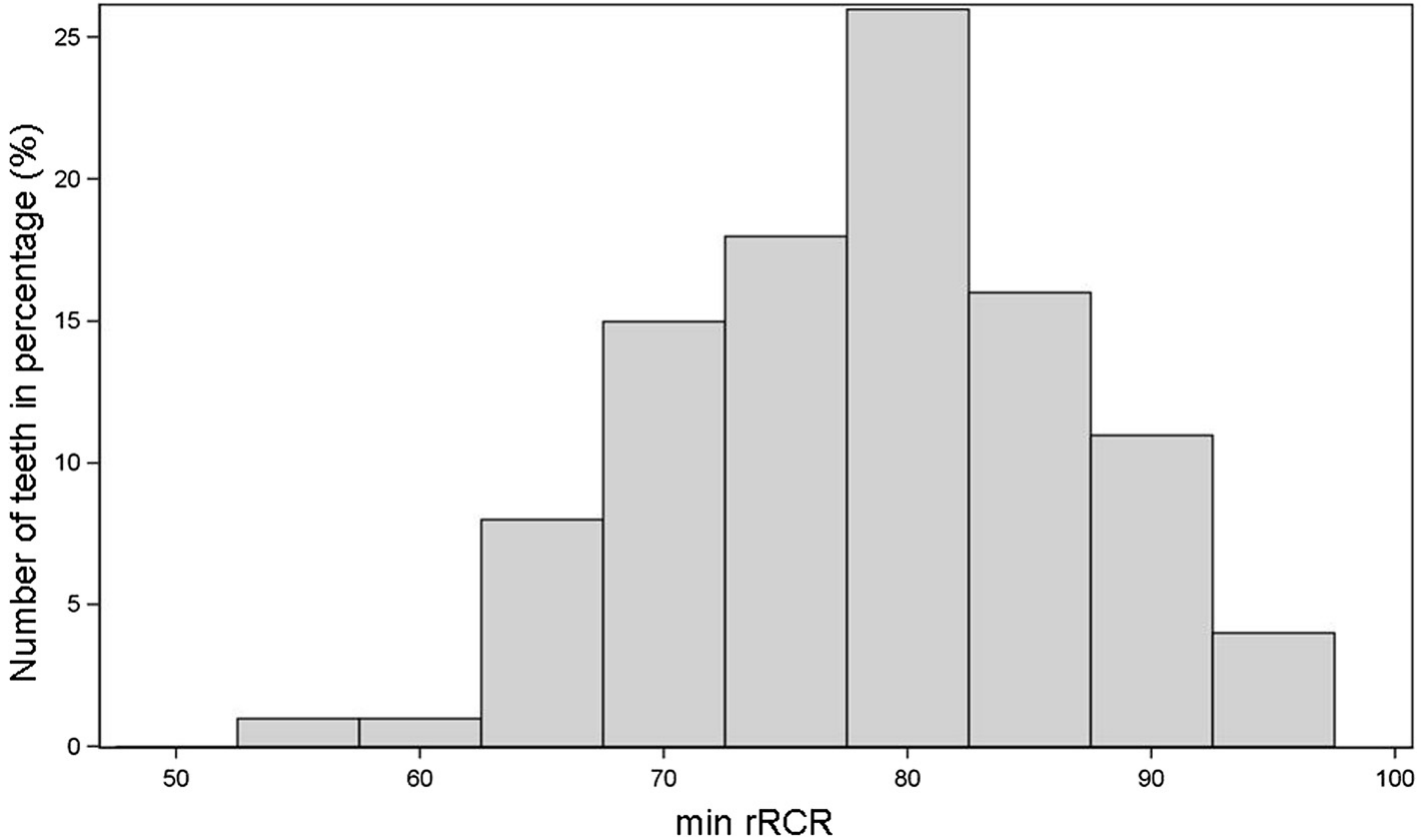
Distribution of the maximum root length reduction, i.e. minimum rRCR (= min rRCR), of each individual tooth (n = 1600).

46% of the 1600 teeth (n = 736) presented a reduction of the post-treatment root length (rRCR < 100%). Considering the severity, a reduction of >0% up to 10% was found in 27.75% (n = 444), a distinct reduction of >10% up to 20% in 11.94% (n = 191) of the sample. 6.31% (n = 101) of the teeth were affected with a considerable reduction (>20%) (Table 3). The values of the individual tooth (tooth 16, 13, 12, 11, 21, 22,-23, 26, 36, 33, 32, 31, 41, 42, 43, 46; each n = 100) are shown in Table 4. To evaluate the extent of the maximum root length reduction during treatment, the distribution of the minimum rRCR values of all teeth are shown in Figure 3. On average, the minimum rRCR was 78.26%. Only a few teeth were affected with a severe reduction of the root length.

**Table 3:** Distribution of the root length reduction during orthodontic treatment: post-treatment RCR relative to pre-treatment RCR (rRCR), absolute (n) and relative (%) frequencies (n = 1600): rRCR < 80 means a reduction over 20% of pre-treatment root length

**rRCR**	**Frequency**	**Percent**
	**(n)**	**(%)**
rRCR < 80	101	6.31
80 ≤ rRCR < 90	191	11.94
90 ≤ rRCR < 100	444	27.75
rRCR ≥ 100	864	54.00

**Table 4:** Distribution of the root length reduction during orthodontic treatment of the individual teeth (upper and lower incisors, canines, first molars) (each n = 100, overall n = 1600): post-treatment RCR relative to pre-treatment RCR (rRCR), absolute (n) and relative (%) frequencies

	**Tooth**															
rRCR	*16*	*13*	*12*	*11*	*21*	*22*	*23*	*26*	*46*	*43*	*42*	*41*	*31*	*32*	*33*	*36*
rRCR < 80	7	12	6	10	7	2	7	8	1	2	1	10	14	5	6	3
	7.0	12.0	6.0	10.0	7.0	2.0	7.0	8.0	1.0	2.0	1.0	10.0	14.0	5.0	6.0	3.0
80 ≤ rRCR < 90	11	11	15	11	12	11	16	8	9	9	18	15	12	11	7	14
	11.0	11.0	15.0	11.0	12.0	11.0	16.0	8.0	9.0	9.0	18.0	15.0	12.0	11.0	7.0	14.0
90 ≤ rRCR < 100	22	24	28	22	22	29	27	36	38	31	25	27	25	32	24	33
	22.0	24.0	28.0	22.0	22.0	29.0	27.0	36.0	38.0	31.0	25.0	27.0	25.0	32.0	24.0	33.0
rRCR ≥ 100	60	53	51	57	59	58	50	48	52	58	56	48	49	52	63	50
	60.0	53.0	51.0	57.0	59.0	58.0	50.0	48.0	52.0	58.0	56.0	48.0	49.0	52.0	63.0	50.0
Total (n)	100	100	100	100	100	100	100	100	100	100	100	100	100	100	100	100

## Discussion

About the incidence of ARR in patients treated with aligners, there is only few reported evidence [3,16]. Therefore, this is the first study, investigating the incidence and severity of ARR in patients with a fulfilled aligner therapy.

ARR of upper incisors during treatment with aligners in a 25-year-old patient were described in a case report [16]. Barbagallo et al. [17] investigated the effects of removable thermoplastic appliances (TA) vs. light or heavy forces with fixed appliances in premolars, which were afterwards extracted. They found that teeth, which experienced orthodontic tooth movement, had significantly more RR than the control teeth. TAs had similar RR as light (25 g) orthodontic forces [17]. But the treatment duration was only eight weeks and only sagittal movements were conducted. In the animal experimental study of Sombuntham et al. [18] clear plastic appliances were inserted but bonded to the residual dentition in rats. Their findings indicated initial superficial RR similar to RR in another group of rats, induced by a closed coil spring. Due to the partly bonding, this force application might not be directly comparable with sequential removable appliances in humans [18]. Thermoplastic aligners are commonly removed several times a day e.g. food consumption.

Practically all patients had ARR. Every patient had a minimum of two teeth affected with a reduction of the root length after treatment. Two recent studies also investigated ARR in patients with a class I occlusion and anterior crowding, but treated with multibracket appliances. They assessed RR with bone beam computed tomography (CBCT) and reported about similar results as the present study [11,12]. 46% of all teeth in our study presented a root reduction. This is in concordance with Castro et al. [11], who reported also about an incidence of 46%. Lund et al. [12] instead described that 91% of all teeth showed some degree of ARR (< 1 mm). This may due to the fact, that the patients in their study were treated with an extraction of one premolar in each jaw quadrant for resolving the anterior crowding. Therefore, the treatment was more extensive than in our study or Castro et al. [11], where no extraction was performed. For the first time we showed that the incidence of ARR in patients treated with aligners was similar to previous studies analyzing the incidence of ARR in patients with multibracket-appliances when resolving anterior crowding.

Regarding influence factors, we found no statistically significant association between the relative changes of RCR and age and gender, which is in concordance with Castro et al. [11]. An association between the root length reduction and the individual tooth could also not be found. Other studies instead reported about most significant ARR and highest frequencies in incisors and first molars. Commonly, maxillary incisors develop ARR more often and more severely than other teeth [6-12]. We found a statistically higher RR in maxillary incisors after extrusion but the value was clinically negligible due to the small mean value.

We also observed no statistical correlation to sagittal and vertical orthodontic tooth movement, except for extrusion of upper incisors. A finite element study demonstrated that the highest amount of stress was produces by vertical forces e.g. extrusion in the apical region [21]. A previous study investigating intrusive and extrusive force applications found that intrusive forces significantly increased RR rates compared with the controls and extrusive forces were not significantly different [22]. In treatments with removable aligners vertical movements are more difficult to perform [19,20,23]. Therefore, they have to be conducted with attachments or other auxillaries like elastics. It could be assumed that those force applications are not very precisely to apply, what might be a reason for causing RR, or the magnitude of the applied extrusive forces was too high. But it should be investigated in a greater sample for more details.

Regarding the clinical relevance of RR in the apical region, the working group of Kalkwarf et al. [24] described that a loss of alveolar bone in the marginal area, caused by periodontal diseases, has more influence on the periodontal support than ARR. Even with an ARR of 3 mm the periodontal attachment still remained 87.10% [24]. They reported that 3 mm of ARR were nearly equivalent to 1 mm of marginal bone loss [24]. Therefore, with regards to the small amount (average reduction of the root length of 4%) and the possibility of being an artefact, the factor extrusion was considered as not clinical relevant.

The first molars, which were less intentionally moved, had also a reduction of the root length. This is in concordance with Castro et al. [11], who also found ARR in molars, when resolving anterior crowding patients with a class I occlusion (using multibracket appliances). Therefore, we concluded that using teeth as an anchorage e.g. with attachments or even being included in the arch where an aligner is inserted might deliver enough force to cause RR.

To measure and assess RR, 2-dimensional radiographs are still presently a common method [13,25]. A striking limitation of this method is that only ARR can be measured. 3-dimensional techniques instead obtain more accurate measurements and give the opportunity to assess the entire root length 3-dimensionally. The degree of ARR seems to be underestimated in panoramic radiographs compared to CBCT [9], when using a scoring system like Levander and Malmgren [9]. Instead of measuring metrically the tooth length in 2-dimensional panoramic radiographs many studies classified changes of the root length in resorption stages [13,26]. We evaluated the relative change of RCR in percentage. RCR was considered stable, even if the two measured radiographs of each patient have different scales (projection-related errors) [7].

In recent studies, the higher reliability and accuracy of CBCT revealing even small amounts of ARR are shown [11,12]. But still, CBCT have not replaced panoramic radiographs, which remains the primary imaging modality. CBCTs have a higher radiation exposure with a greater medical risk for the patient [27]. The effective doses of CBCTs can be 1.5 to 33 times higher than panoramic radiographs [28].

A limit of this investigation is the fact that it is a retrospective study. But due to the stringent inclusion criteria, the fact that all patient were treated by the same practitioner, and all radiographs taken by the same device, offers this study reliable results with a high number of patients.

## Conclusion

The present study is the first analyzing apical roots resorptions in patients with a fully implemented orthodontic treatment with aligners. Every patient had a minimum of two teeth with root length reduction when resolving anterior crowding. On average, 7.36 teeth per patient were affected. Overall, 54% of the measured 1600 teeth showed signs of apical root resorption, 6.31% a reduction of over 20% of the pre-treatment root length. No clinical relevant influence factor could be detected. Therefore, due to the high individual variability in the degree of the occurred root resorptions, there is no prediction by external influence factors.

## Abbreviations

ARR: Apical root resorption; RR: Root resorption; RCR: Root-crown-ratio; rRCR: Relative root-crown-ratio.

## Competing interests

The authors declare that they have no conflict of interest.

## Authors’ contributions

EK carried out the conception of the study. She supervised the measurements, assembled the data, conducted the interpretation of the data, and drafted the manuscript. TD provided the patient pool from which the sample was selected. IS analyzed the data and helped with the manuscript. CJ was involved in conception and design of the study, also in analysis and interpretation of data, and drafting the manuscript. SH collected the data and conducted the measurements. HW conceived of the study, and participated in its design and coordination and helped to draft the manuscript. All authors read and approved the final manuscript.
